# Seeing is believing: an educational outreach activity on disinfection practices

**DOI:** 10.1186/1477-7517-5-7

**Published:** 2008-02-12

**Authors:** Sarah-Amelie Mercure, Isabelle Tetu, Steeve Lamonde, Francoise Cote, Guides de rue working group

**Affiliations:** 1 Faculté des Sciences infirmières, Université Laval, Pavillon Agathe-Lacerte, Québec (Qc), Canada; 2 Programme interfacultaire en Santé Communautaire, Université Laval, Québec (Qc), Canada; 3 Point de Repères, 530 Saint-Joseph est, Québec (Qc), Canada

## Abstract

**Background:**

Skin and soft-tissue infections are very common among persons who inject drugs. They occur when microbes pass under the protective layer of the skin and proliferate. This happens when harm reduction recommendations such as skin aseptia before injection and sterile injection equipment usage are not properly followed.

**Methods:**

A group of active drug users involved in a health promotion project as peer educators were asked about their formation needs. To address their inquiries concerning skin and soft-tissue infections, we devised with them a series of workshops touching upon common infections, the microflora, and microbial transmission.

**Results:**

Participants learned to identify common infections and how to properly react in case of an abscess, cellulitis or phlebitis. They saw microscopic objects, found out about the high prevalence of microbes in their environment and on their skin, and could appreciate the efficiency of different washing and disinfection techniques. They visualized how easily microbes can spread from person to person and from contaminated objects to persons.

**Conclusion:**

In the weeks following this activity, some participants demonstrated and reported healthy behavioural changes regarding their own injection practices. Furthermore, they shared their newfound knowledge and began enforcing its application among people they inject drugs with. Most participants greatly appreciated this activity and valued it as being highly efficient and tangible. Note: A French version of this paper is available on the *Journal*'s web site [see Additional file 1].

## Background

Skin and soft-tissue infections such as abscesses and cellulitis are some of the most common cause of emergency room visits among people who inject drugs [1-3]. These may occur when usual harm reduction recommendations, such as unique usage of syringes and skin aseptia before injection [4], are not properly and consistently followed. Based on their very high prevalence rates [5], some users perceive soft-tissue infections as normal and somehow inevitable consequences of injection [6].

As they cause pain and can lead to potentially life threatening conditions [7,8], these consequences of unsafe injection practices are of major concern for community health workers who intervene with them [9].

We report of an educational activity which aim was to address the formation needs expressed by a group of peer educators regarding skin and soft-tissue infections. The objectives were to sensitize them to the prevalence, spread and potential harm of microbes in the environment and on the skin, and to verify how efficient their current disinfection practices were. The results of a short-term formative evaluation are presented.

## Methods

### Participants

Participants for this activity were recruited for their motivation to become health advocates among their peers and their large social network. The 17 persons appointed for the activity were current members of a peer-based intervention (see Appendix 1 for more information) [10]. Of the 17 persons invited to participate, 11 showed up. Six males and five females took part in the workshops described here. These persons reported that they typically met between 3 and 200 different injection drug users each week (median: 12 persons), were 37 to 57 years old (median: 47 years old), and began injecting drugs 3 to 43 years ago. One of them was a former drug user, and other participants were active users of cocaine (n = 8) or opiates (n = 2). As is the case for all activities of the peer intervention project, a 20$-stipend was offered to participants. This project was approved by the ethics committee of Université Laval. Participants provided informed consent.

### Description of the activity

Three workshops were held simultaneously, with small groups of three or four persons attending all of the 30-minute workshops alternately. These workshops are briefly described here (more detailed description available upon request). One workshop was facilitated by a community health nurse and touched upon the identification of common injection-related skin infections and their complications if not properly treated. In the same workshop, participants were asked to show how they bleached their used syringes. Two participants per group performed the behaviour according to their own standards and based on suggestions of other participants [see Additional file 2].

A second workshop, entitled 'Microbes around us', focussed on showing the ubiquity of micro-organisms in the environment and on the skin, as well as on the relative efficiency of different washing and disinfection methods. This workshop was prepared and facilitated by a graduate microbiology student. Briefly, a microscope was mounted on a TV set, and samples were taken and displayed so that participants could actually *see *microscopic objects [see Additional file 3]. They then were asked to inoculate an agar plate (tryptone bile agar) (1) with their unwashed finger, (2) after washing their hands with antibiotic soap and warm water for 30 seconds, and (3) after rubbing their finger with an alcohol swab. They were instructed to use the swab as they usually did. Samples were also taken from their cubital fossa, lips, tongue, and they were asked to cough onto a plate [see Additional file 4]. Participants were invited to come back three days later to view the results. Five of them accepted this invitation.

For the third workshop, the outreach worker who recruited the participants and who keeps weekly contacts with them illustrated how easily microbes can be transmitted. He used the Glo-Germ^® ^kit following manufacturer's recommendations (Glo-Germ Product Co, Moat, UT, USA) [see Additional file 5]. Briefly, after putting a UV-inducible fluorescent powder on his hands and informally shaking hands with participants, he asked them to put their hands under a UV-lamp to reveal the fluorescence. He then asked them to wash their hands as usual and to return to see if all the fluorescence had disappeared, focussing their observations on nails and cracks of the skin. He then demonstrated the proper hand washing technique, and participants were asked to practice it and to visualize the results under the UV-lamp.

### Data collection

After incubation for three days at 37°C, agar plates were scored visually for abundance and diversity of microbes. They were photographed and properly disposed of thereafter. An information sheet to be distributed and explained to all peer educators was then produced based on the pictures. Participants' reactions to the content/results of the workshops were collected by means of (1) an anonymous self-administered satisfaction questionnaire, and (2) a group discussion held a month later. The questionnaire was filled immediately following the activity and contained four sections. The first three sections addressed participation issues (3 items; e.g. I participated actively in small group discussions), knowledge (8 items; e.g. I know better about skin infections such as abscesses and cellulitis), and overall satisfaction (3 items; e.g. I appreciated this training session). They were answered according to a 5-level scale (not at all to a lot). The fourth section was dedicated to comments (open-ended). The group discussion held a month later was a "*rencontre bilan*" (meeting to take stock of progress). With participants' consent, it was recorded for research purpose. Many topics were discussed and those touching upon the workshops presented in this paper were used for data analysis.

### Data analysis

Bacterial count and diversity data were analysed in order to verify whether the median change in bacterial scores before and after hand washing and alcohol rubbing significantly differed from zero. As samples were non-independent and not normally distributed, the Wilcoxon signed-rank test was used in these calculations [11]. Results of the satisfaction questionnaire are reported as frequency items. The group discussion was transcribed verbatim and data touching upon the workshops presented here were extracted. Representative quotations are presented. Statistical analyses were carried out using the SAS software version 9.1.

## Results

Our satisfaction survey showed that all participants but one rated their overall appreciation of the activity at the highest level. Afterwards, they felt they were better or much better able to expose their ideas, and all but one participant had the strongest intention to participate in further project activities.

### Workshop 1: common infections and bleach used

After completing this workshop, all participants but one felt fairly (n = 6) or much more (n = 4) able to refer someone having a skin infection in a timely manner. The majority felt much better able to give good advices to someone having injection-related skin infection. All participants agreed they acquired enough (n = 3) or lots (n = 8) of knowledge touching upon soft-tissue complications such as abscesses, cellulitis and phlebitis. Weeks after the workshop, some participants kept contact with the nurse and asked her to validate their putative skin infections identification. For example, one participant could accurately identify her boyfriend's cellulitis at an early time-point and reacted accordingly (i.e. cold compress application and medical consultation for antibiotics, [12]).

Regarding bleach use, none of the participants displayed the proper technique. They did not wait long enough (typically much less than the usually recommended 30 seconds, [13]), neither did they rinse the syringe with clean water before and after rinsing it with bleach. They reported that they did not know if they had to use it diluted or pure, and were not knowledgeable of the proper way to store bleach (i.e. with the cap on to prevent evaporation of chlorine). Interestingly, before the workshop, they believed that a bleached syringe was a sterile syringe. Judging by their discourse, this perception changed following the activity. After the workshop, participants knew better (n = 3) or much better (n = 8) the limits associated with bleach use.

### Workshop 2: microbes around us

**Figure 1:**
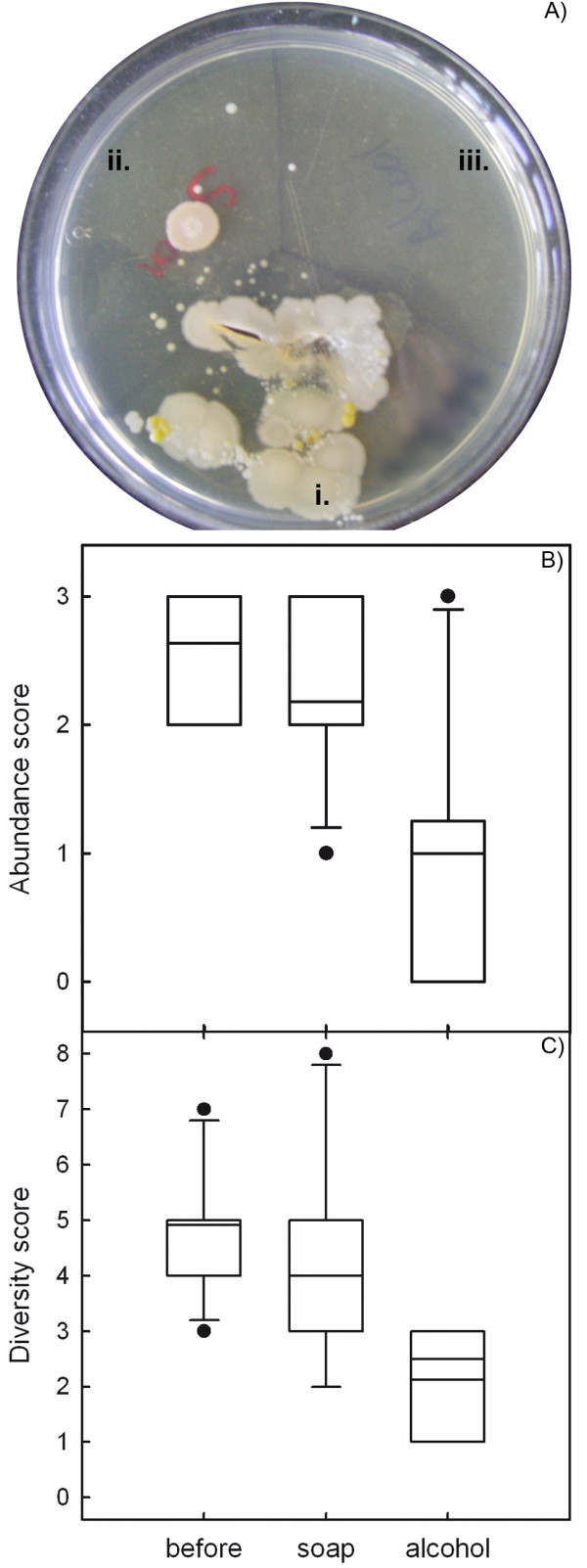
**Microbes of the skin**. Agar plates were inoculated with participants' thumb i) before washing it, ii) after washing it with antibiotic soap and warm water for 30 seconds, and iii) after rubbing with an alcohol swab. A) A plate where alcohol was used efficiently. B) Box plots showing microbial abundance computed as a categorical score (3:>300 colonies, 2:50–300 colonies, 1:<50 colonies). Abundance did not significantly differ after washing with soap (*p *= 0.13), and was reduced by alcohol rubbing following hand washing (*p *= 0.03). C) Microbial diversity expressed as the number of visually differing colonies. Diversity did not significantly differ after washing with soap (*p *= 0.16), and was reduced by alcohol rubbing following hand washing (*p *= 0.02).

Bacterial growth was noted on all plates inoculated with unwashed and washed hands, and on eight out of eleven plates inoculated with alcohol-rubbed fingers (Fig. 1A). In our experimental conditions, casual hand washing with antibiotic soap and warm water did not significantly reduce bacterial abundance and diversity (Wilcoxon signed-rank test, *p *> 0.13), whereas rubbing fingers with an alcohol swab after washing them with soap significantly reduced both bacterial growth and diversity (*p *< 0.03) (Fig. 1B,C). After this workshop, participants rated their knowledge about alcohol action on microflora as better (n = 1) or much better (n = 10) than before the activity.

Samples taken from cubital fossa, lips and tongue were, as expected, highly colonised with microbes. Cough plates were used to illustrate how air is a potential source of inoculums. Along with the lips and tongue plates, they also constituted tangible evidence against the practice of licking a needle before inserting it into a vein. After the workshop, participants thus felt they knew better (n = 3) or much better (n = 7) how microbes of the normal microflora were likely to cause infections when introduced under the skin (1 missing datum).

### Workshop 3: transmission

Fluorescence was seen by all participants whose hands came in contact with the 'source', either via hand shaking or through manipulation of objects previously handled by the source. Usual hand washing was generally not sufficient to remove all of the fluorescent powder. After this workshop, participants perceived they knew enough (n = 2) or very well (n = 7) how easily microbes can be transmitted from person to person or from objects to persons (2 missing data).

## Discussion

The simple activity described in this report was motivated by the interest of participants to know more about hygiene and skin/soft-tissue infections. This concern was express by the majority of participants during preliminary work, and they rated it as a priority when they established their 'formation curriculum'. It was also outreach workers' top-priority as revealed before [9].

While our data do not provide evidence for the efficacy of the educational activity in reducing injection-related harm, they confirm that the information was well understood by participants. For example, for those who began the activity with the workshop on transmission, it was striking how vigorously they washed hands when they latter attended the 'Microbes around us' workshop. Visualising the effect of proper and improper hand washing was an incentive to instantly adjust their behaviour. As one participant said, "We're finally shown what we're doing wrong!"

How the peer educators will use this first-hand information and transform it into knowledge from which to take health-related decisions, however, is more speculative. We are aware of one participant, a shooting gallery manager, who posted pictures of the agar plates with bacterial growth on his wall to discuss the results with his clients. He enforced a new "law": at his place, no one will use *any *injection material more than once.

This example provides support for the strategies we used to help key members of the community building their capacities for health promotion. These strategies relied on behavioural implication of the participants. As they stated, they are often surrounded with harm reduction information, but this information does not always affect their behaviours. In fact, it is noteworthy that none of the participants, some of which had been injecting for several decades, displayed the proper bleaching technique, and that they all stated they learned new information about the use of alcohol for skin aseptia. Having an opportunity to experience with a behaviour, to practice it after observing a model, and to gauge its effects might thus prove an interesting way to induce behavioural change among this community.

## Conclusion

Our paper describes an educational intervention designed to reduce the adverse medical consequences associated with drug injection. To do so, we established a partnership with community members willing to help their peers. We worked in collaboration with those key members of the drug using community in order to fill the gaps they identified in their knowledge and capacities. After completing their self-established curriculum, their aim is to help other drug users in a harm reduction perspective.

Our most important finding is that it is possible to organize successful workshops with persons who actively use injection drugs. In line with previous recommendations and reports [14], we corroborate that working on capacity building with marginalized people is possible and much appreciated by both users and health educators.

From a practical viewpoint, our workshops also demonstrated that important harm reduction messages such as skin cleaning and injection materials disinfection were not fully integrated by participants prior to the activities. The positive behavioural changes some of them reported afterwards suggest that our training approach was adequate. As they thereafter displayed these healthier behaviours when they used drugs with their peers, they likely became models of harm reduction.

Thus, no matter how drug policies change over time and political allegiance in regards to harm reduction, this approach will remain part of our communities' toolbox.

## Competing interests

The author(s) declare that they have no competing interests.

## Authors' contributions

SAM wrote the manuscript and contributed ideas to the design, contents and interpretation of the activity reported. IT contributed ideas, facilitated one workshop and revised the manuscript. SL recruited participants, facilitated one workshop and revised the manuscript. FC is the principal investigator, conceived the study, designed the data collection instruments and was involved in drafting the manuscript. The *Guides de rue *working group contributed ideas to the design and realization of the activity described. All authors read and approved the manuscript.

## Appendix

### Appendix 1 – *Les Guides de rue *(Street Guides)

*Les Guides de rue *is a three-year action-research project (2005–2008). It involves the working together of Québec city's drug using community, Point de Repères (local syringe exchange programme), and researchers from Laval University. The collaboration aims at developing a peer helping network in the community. The project involves two phases: first a capacity-building intervention among peer helpers, then the 'intraventions' of these peer helpers.

The intervention phase started when persons known by outreach workers from Point de Repères to be interested in helping others were approached by the project's staff. They were interviewed and invited to participate. Seventeen persons made this commitment. Their first task was to identify their educational needs. They then sorted those needs in decreasing order of importance. The consensus they reached was as follows: 1) first aid and CPR in case of an overdose, 2) counselling techniques, 3) skin care and best practices to avoid skin infections, 4) legal aspects touching upon peer helping, 5) resources available in the community for persons who inject drugs, 6) effects of drugs (especially new drugs and their interactions), 7) how to manage your place, and 8) HIV and HCV.

All these topics were then touched upon in a series of 8 consecutive workshops. The workshops, held on a monthly basis, were co-prepared by 2–3 members of the group who were interested in a given topic, the project's outreach worker, and 2–3 members of the research staff. It had to be practical, accessible and evidence-based. The format was adjusted to the lifestyle and needs of participants. For that purpose, workshops were always preceded by a warm meal, they were held monthly from 5 p.m. to 7 p.m., and the contents covered were summarized in hand-outs distributed to each participant.

This first capacity-building phase just ended and we are now in the 'intravention' part of the project. Peer helpers now use their newly developed skills and knowledge to interact with their peers in the community settings. They do so at their own pace and adopt a harm reduction approach. They still keep regular contacts with the project's outreach worker.

## Supplementary Material

Additional File 1 Article en Français (article in French). Une version française de l'article a été préparée par les auteurs. Elle est disponible à partir du site Web du *Harm Reduction Journal*. Click here for file

Additional File 2 Workshop 1 (photograph). A participant is demonstrating the way she uses bleach to clean a syringe and needle as she usually does when she does not have access to sterile material. Click here for file

Additional File 3 Workshop 2, part 1 (photograph). A microscope was mounted on a TV-set so that participants could see microscopic objects sampled from the surroundings. Click here for file

Additional File 4 Workshop 2, part 2 (photograph). **A**) A participant is taking a sample from her cubital fossa. **B**) Microbial growth from this sample. Click here for file

Additional File 5 Workshop 3 (photograph). **A**) The set-up used to demonstrate hand washing techniques. **B**) A participant's hands under the UV-lamp. Some fluorescent powder remained after he washed his hand as can be seen on the tips of his fingers. Click here for file
