# Supplementary material for: Seeing is believing: an educational outreach activity on disinfection practices
Source: Harm Reduct J. 2008 Feb 12;5:7. doi: 10.1186/1477-7517-5-7 (PMC2265699; doi:10.1186/1477-7517-5-7)
Supplement: Additional File 1 — Article en Français (article in French). Une version française de l'article a été préparée par les auteurs. Elle est disponible à partir du site Web du Harm Reduction Journal. [file 1477-7517-5-7-S1.doc]

# Le voir pour le croire : une activité éducative sur les pratiques de désinfection de la peau

Sarah-Amélie Mercure^1,2^, Isabelle Têtu^2,3^, Steeve Lamonde^3^, Françoise Côté^1,2§^, le groupe des *Guides de rue*

^1^Faculté des Sciences infirmières, Université Laval, Pavillon Agathe-Lacerte, Québec (Qc), Canada

^2^Programme interfacultaire en Santé Communautaire, Université Laval, Québec (Qc), Canada

^3^Point de Repères, 530 Saint-Joseph est, Québec (Qc), Canada

^§^Auteure de correspondance

Addresses de courriel:

SAM: [sarah-amelie.mercure.1@ulaval.ca](mailto:sarah-amelie.mercure.1@ulaval.ca)

IT: [isatetu@hotmail.com](mailto:isatetu@hotmail.com)

SL: [pointderepere@qc.aira.com](mailto:pointderepere@qc.aira.com)

FC: [francoise.cote@fsi.ulaval.ca](mailto:francoise.cote@fsi.ulaval.ca)

*Guides de Rue*: [guidesderue@hotmail.com](mailto:guidesderue@hotmail.com)

# Résumé

## Contexte

Les personnes qui s’injectent des drogues présentent souvent des infections de la peau et des tissus mous. Ces infections se développent lorsque des microbes traversent la couche protectrice de la peau et prolifèrent. Ces infections peuvent se produire quand les recommandations de réduction des méfaits comme l’asepsie avant l’injection et l’utilisation de matériel stérile ne sont pas correctement appliquées.

## Méthode

Des personnes consommatrices de drogues injectables impliquées dans un projet de pairs aidants ont été interrogées quant à leurs besoins de formation. En réponse à leurs demandes concernant les infections de la peau et des tissus mous, nous avons préparé une co-formation regroupant trois thèmes : les infections les plus communes, la flore microbienne et les modes de transmission des microbes.

## Résultats

Les participants ont appris à identifier les infections les plus communes et à réagir de manière appropriée en présence d’un abcès, d’une cellulite ou d’une phlébite. Ils ont également pu observer des objets microscopiques, découvrir la forte prévalence de microbes dans leur environnement ainsi que sur leur peau et apprécier l’efficacité de différentes techniques de lavage des mains et de désinfection. Finalement, ils ont visualisé la manière dont les microbes peuvent être transmis d’une personne à une autre et d’un objet contaminé à une personne.

## Conclusions

Dans les semaines qui ont suivi cette activité, plusieurs participants ont changé certains de leurs comportements d’injection et ont rapporté ces changements auprès du groupe. Certains ont partagé leur nouveau savoir avec d’autres personnes avec qui ils s’injectent et ont commencé à promouvoir son application auprès de ces personnes. La plupart des participants ont grandement apprécié cette activité et l’ont trouvée efficace et concrète. Note : La version anglaise originale de cet article est disponible sur le site Web du *Harm Reduction Journal*.

# Introduction

Les infections de la peau et des tissus mous tels les abcès et cellulites sont l’une des causes majeures des visites aux urgences pour les personnes qui s’injectent des drogues [1-3]. Ces infections se produisent lorsque certaines recommandations de réduction des méfaits comme l’utilisation d’une seringue unique et l’asepsie de la peau avant l’injection [4] sont appliquées incorrectement ou de manière intermittente. En raison de leur forte prévalence [5], plusieurs utilisateurs perçoivent ces infections comme des conséquences normales et inévitables de leur mode de consommation [6].

Comme elles causent de la douleur et peuvent même être fatales [7, 8], les conséquences des pratiques d’injection non sécuritaires constituent une préoccupation majeure pour les travailleurs communautaires qui interviennent avec les personnes qui s’injectent des drogues [9].

Dans cet article, nous rapportons une activité éducative dont le but était de répondre aux besoins de formation exprimés par un groupe de pairs éducateurs à propos des infections de la peau et des tissus mous. Les objectifs étaient de les sensibiliser à la prévalence, la transmission et les dangers potentiels des microbes dans leur environnement et sur leur peau, ainsi que de vérifier l’efficacité de leurs pratiques actuelles de désinfection. Les résultats d’une évaluation formative de cette formation sont présentés.

# Méthode

*Participants*. Les participants à cette activité ont été recrutés pour leur motivation à devenir des promoteurs de la santé parmi leurs pairs et en raison de leur réseau social important. Les 17 personnes conviées à l’activité étaient des membres réguliers d’un projet d’intervention par les pairs (voir l’Appendice 1 pour plus de détails) [10]. Parmi ces personnes, 11 se sont présentées à l’activité. Six hommes et cinq femmes ont ainsi pris part aux ateliers décrits ci-après. Ces participants rapportaient rencontrer entre 3 et 200 différentes personnes s’injectant des drogues chaque semaine (médiane : 12 personnes), ils avaient entre 37 et 57 ans (médiane : 47 ans) et s’injectaient depuis 3 à 43 ans. Un participant était un ancien utilisateur, alors que les autres étaient des consommateurs actifs de cocaïne (n = 8) ou d’opiacés (n = 2). Comme pour l’ensemble des activités de formation du projet, une compensation de 20$ était offerte aux participants. Ce projet a été approuvé par le Comité d’éthique de l’Université Laval et les participants ont fourni un consentement éclairé.

*Description de l’activité*. Trois ateliers se tenaient simultanément. Par petits groupes de trois ou quatre, les participants se rendaient à tour de rôle dans chacun des ateliers qui duraient en moyenne 30 minutes chacun. Ces trois ateliers sont brièvement décrits dans ce qui suit (plus de détails sont disponibles sur demande). Un premier atelier était animé par une infirmière communautaire. Il s’intéressait à l’identification des infections cutanées les plus courantes ainsi qu’à leurs complications possibles advenant une prise en charge inadéquate. Dans cet atelier, les participants étaient également invités à faire une démonstration de la manière dont ils utilisaient l’eau de javel pour désinfecter leurs seringues usagées. Deux personnes par groupe faisaient l’exercice et pouvaient demander conseil aux autres participants.

Un second atelier, intitulé les *Microbes autour de nous*, permettait aux participants d’apprécier l’ubiquité des microorganismes présents dans l’environnement et sur la peau et permettait de montrer l’efficacité relative de différentes méthodes de lavage des mains et de désinfection. Cet atelier a été préparé et animé par une étudiante graduée en microbiologie. En bref, un microscope était branché sur un téléviseur et des échantillons étaient prélevés puis affichés à l’écran pour que les participants *voient* des objets microscopiques. Ils étaient ensuite invités à inoculer un pétri d’agar (tryptone bile agar) (1) avec leur doigt non lavé, (2) après avoir lavé leurs mains avec un savon antibiotique et de l’eau tiède pendant 30 secondes et (3) après avoir nettoyé leur doigt avec un tampon d’alcool. Les consignes fournies étaient d’utiliser le tampon d’alcool selon leur routine habituelle. Des échantillons ont aussi été prélevés dans le pli du coude, sur les lèvres et sur la langue. On leur demandait également de tousser à la surface d’une gélose. Les participants étaient par la suite invités à revenir trois jours plus tard pour voir les résultats. Cinq d’entre eux ont accepté l’invitation.

Pour le troisième atelier, le travailleur de rue qui avait recruté les participants et qui garde un contact hebdomadaire avec eux a illustré la manière dont les microbes peuvent facilement être transmis. Pour ce faire, il a utilisé l’ensemble Glo-Germ® en suivant les recommandations du manufacturier (Glo-Germ Product Co, Moat, UT, USA). Brièvement, après avoir enduit ses mains de poudre fluorescente et serré la main des participants de manière informelle, il leur a demandé de les placer sous la lampe UV. Par la suite, il leur a demandé de se laver les mains selon leur routine habituelle puis de retourner voir sous la lampe pour vérifier si la fluorescence avait disparu. Il leur a suggéré de bien observer sous leurs ongles et au niveau des plis cutanés. Il a ensuite démontré la manière appropriée de se laver les mains, puis les a invités à la pratiquer avant de procéder à une nouvelle observation sous la lampe UV.

*Collecte des données*. Après trois jours d’incubation à 37°C, les pétris d’agar ont été examinés pour évaluer l’abondance et la diversité des microbes. Avant d’en disposer de manière appropriée, ils ont été photographiés. Sur la base de ces images, un court document a été produit pour expliquer aux pairs éducateurs la signification des images. Leurs réactions au contenu et aux résultats des ateliers ont été collectées de deux façons : (1) un questionnaire de satisfaction auto administré et anonyme et (2) un groupe de discussion tenu un mois plus tard. Le questionnaire était complété immédiatement après les activités et contenait quatre sections. Les trois premières sections s’intéressaient au niveau de participation à l’atelier (3 items; e.g. J’ai participé activement aux petits groupes de discussion), aux connaissances acquises (8 items; e.g. Je connais mieux les infections de la peau comme les abcès et les cellulites) et au niveau de satisfaction générale (3 items; e.g. J’ai apprécié cette formation). Chacune de ces questions se répondait sur une échelle à cinq niveaux (i.e. pas du tout, peu, plus ou moins, assez, beaucoup). La quatrième section était réservée pour des commentaires. Quant au groupe de discussion, il s’agissait d’une « rencontre bilan » qui récapitulait l’ensemble des formations dispensées depuis le début du projet. Avec le consentement des participants, les échanges ont été enregistrés. Plusieurs sujets ont été abordés et ceux touchant les ateliers présentés dans cet article ont été inclus dans l’analyse des données.

*Analyse des données*. Le décompte bactérien et les données de diversité ont été analysés pour vérifier si les valeurs médianes avant et après le lavage des mains et l’utilisation de l’alcool étaient significativement différentes. Comme l’échantillon était non indépendant et anormalement distribué, le test de Wilcoxon a été utilisé [11]. Les résultats du questionnaire de satisfaction sont rapportés en termes de fréquence des réponses aux différents items. L’enregistrement du groupe de discussion a été transcrit *verbatim* et les données se rapportant à l’atelier ont été analysées. Des extraits représentatifs sont présentés. Les analyses statistiques ont été menées avec le logiciel SAS version 9.1.

# Résultats

Les résultats du questionnaire auto rapporté indiquaient que 10 des 11 participants avaient apprécié au plus haut point cet atelier. Suite à cette rencontre, ils se disaient plus confiants en leurs capacités à exposer leurs idées. Tous sauf un ont exprimé leur intention ferme de participer aux prochaines activités du projet.

## Atelier 1 – les infections communes et l’utilisation de l’eau de Javel

Après avoir complété cet atelier, tous les participants sauf un se sentaient assez (n = 6) ou beaucoup (n = 4) plus capables de référer quelqu’un qui présenterait une infection de la peau et ce, en temps opportun. La majorité se percevait plus à même de donner des conseils à un pair en lien avec les infections de la peau. Tous les participants s’accordaient pour dire qu’ils avaient acquis assez (n=3) ou beaucoup (n=8) de connaissances concernant les abcès, cellulites et phlébites. Plusieurs semaines après l’atelier, certains participants ont gardé contact avec l’infirmière communautaire et lui ont demandé de valider leur identification d’infections de la peau. À titre d’exemple, une participante a pu identifier la cellulite de son conjoint à un stade précoce et a réagi en conséquence (i.e. application de compresses froides et consultation médicale pour antibiothérapie [12]).

Concernant l’utilisation de l’eau de Javel, aucun participant n’avait la bonne technique. Ils n’attendaient pas assez longtemps (beaucoup moins que les 30 secondes généralement recommandées [13]) ni ne rinçaient leur seringue à l’eau claire avant et après avoir utilisé l’eau de Javel. Ils ne savaient pas s’il fallait l’utiliser concentrée ou diluée, pas plus qu’ils n’avaient connaissance de la manière adéquate de la conserver (i.e, avec le capuchon pour prévenir l’évaporation du chlore). Il est intéressant de constater qu’avant l’atelier la majorité des participants croyait qu’une seringue nettoyée à l’eau de Javel devenait une seringue stérile. À en juger par leur discours, cette perception a été modifiée suite à l’atelier. Après cette formation, les participants connaissaient mieux (n = 3) ou vraiment mieux (n = 8) les limites associées à l’usage de l’eau de Javel.

## Atelier 2 – les microbes autour de nous

Une croissance bactérienne a été notée sur toutes les géloses inoculées par des mains lavées ou non et sur huit des onze géloses inoculées avec des doigts frottés à l’alcool (Fig. 1A). Dans nos conditions, le lavage des mains avec un savon antibiotique et de l'eau tiède n'a pas réduit de manière significative l'abondance et la diversité bactériennes (Wilcoxon signed-rank test, *p*>0.13), alors que la désinfection des doigts avec un tampon d’alcool, précédée d’un lavage au savon, réduisait significativement la croissance bactérienne et la diversité (*p*<0.03) (Fig. 1B, C). Suite à cet atelier, les participants ont rapporté que leurs connaissances quant à l'action de l'alcool sur la microflore s’étaient assez (n = 1) ou beaucoup améliorées (n = 10).

Tel qu’attendu, les échantillons prélevés au pli du coude, sur les lèvres et sur la langue étaient fortement colonisés par des microbes. Les pétris inoculés par les participants ayant toussé à la surface des géloses ont permis d’illustrer la manière dont l’air peut représenter une source d’inoculum. Avec ceux des lèvres et de la langue, ils constituaient une preuve tangible allant à l’encontre de la pratique répandue consistant à lécher l’aiguille avant de l’introduire dans une veine. Après l'atelier, les participants ont estimé qu'ils connaissaient assez (n = 3) ou beaucoup (n = 7) mieux la manière dont les microbes de la microflore normale peuvent causer des infections lorsqu'ils sont introduits sous la peau (1 donnée manquante).

## Atelier 3 – la transmission des microbes

Tous les participants dont les mains sont venues en contact avec celles de l’animateur ou avec un objet qu’il avait préalablement manipulé ont pu constater la présence de poudre fluorescente sur leurs mains. Le lavage de leurs mains selon leur routine habituelle n’était pas suffisant pour enlever toute la poudre fluorescente. Après l’atelier, les participants percevaient qu’ils en savaient assez (n=2) ou beaucoup (n=7) sur la facilité avec laquelle des microbes sont transmis d’une personne à une autre ou d’un objet à une personne (2 données manquantes).

# Discussion

Les activités simples décrites dans cet article ont été motivées par l’intérêt manifesté par les participants qui voulaient en connaître davantage sur l’hygiène et les infections de la peau et des tissus mous. Ce besoin avait été exprimé par la majorité du groupe lors d’une séance de travail préliminaire. Il avait été jugé prioritaire lors de l’établissement de leur curriculum de formation. Notons qu’il s’agissait également d’un besoin prioritaire pour les intervenants communautaires tel que l’avaient révélé des travaux antérieurs [9].

Bien que nos données n’apportent pas de preuves quant à l’efficacité de ces formations en termes de réduction des méfaits liés aux pratiques d’injection, elles confirment que l’information a très bien été comprise par les participants. Par exemple, nous avons pu constater que ceux qui avaient débuté la rencontre avec l’atelier sur la transmission des microbes se lavaient beaucoup plus vigoureusement les mains que les précédents lors de leur passage dans l’atelier « les microbes autour de nous ». Visualiser l’effet de différentes techniques de lavage des mains était devenu un incitatif instantané pour ajuster leur comportement. Comme l’un des participants le mentionnait, « On nous montre finalement ce qu’on fait de pas correct! ».

La manière dont les participants utiliseront cette information de première main et la transformeront en savoir pour éclairer leurs décisions en matière de santé demeure spéculative. Cependant, nous savons que l’un d’eux, un participant qui reçoit régulièrement des consommateurs à sa résidence, a affiché les images des pétris avec croissance bactérienne pour en discuter avec ses clients. Il a également établi une nouvelle loi chez lui : plus personne n’a le droit d’utiliser le moindre matériel d’injection plus d’une fois.

Cet exemple fournit un soutien pour les stratégies que nous avons utilisées pour aider des membres clés de la communauté à renforcer leurs capacités en matière de promotion de la santé. Ces stratégies sont fondées sur l’implication des participants. Comme ils l’ont indiqué, ils sont souvent entourés d’information sur la réduction des méfaits, mais cette information n’affecte pas toujours leurs comportements. En effet, il est à noter qu’aucun des participants – parmi lesquels certains s’injectaient depuis plusieurs décennies – n’utilisait adéquatement l’eau de Javel. Également, ils ont tous déclaré avoir appris de nouvelles informations à propos de l’usage de l’alcool pour désinfecter leur peau. Avoir l’opportunité d’expérimenter un comportement, de le pratiquer après avoir observé un modèle et de mesurer ses effets pourrait se révéler une manière intéressante d'induire un changement comportemental au sein de cette communauté.

# Conclusions

Ce bref article a décrit une intervention éducative dont l’objectif était de réduire les effets délétères associés à l’injection de drogues. Pour ce faire, nous avons établi un partenariat avec des membres de cette communauté désireux de venir en aide à leurs pairs. Nous travaillons en collaboration avec eux pour combler les lacunes qu’ils ont eux-mêmes identifiées dans leurs savoir et habiletés. Après avoir complété leur cursus de formation, leur but est de venir en aide aux autres personnes utilisant des drogues dans une perspective de réduction des méfaits.

Pour l’instant, notre plus important résultat est de pouvoir affirmer qu’il est possible d’organiser avec succès des ateliers avec des personnes qui consomment activement des drogues injectables. En accord avec d’autres [14], nous corroborons que le travail avec des personnes marginalisées dans une optique de construction des capacités est à la fois possible et fort apprécié, tant par les participants que par les éducateurs.

D’un point de vue pratique, notre activité démontre aussi que d’importants messages de réduction des méfaits comme le nettoyage de la peau et la désinfection du matériel d’injection n’étaient pas complètement intégrés par les participants avant les ateliers. Des changements comportementaux positifs rapportés par certains après les activités suggèrent que notre approche de formation était adéquate. Comme ils appliquent ces comportements sains quand ils utilisent des drogues avec leurs collègues, ils peuvent devenir des modèles pour la réduction des méfaits.

Ainsi, quelle que soit la façon dont les politiques sur les drogues changent avec le temps ou les allégeance politique en regard de la réduction des méfaits, cette approche demeurera dans le coffre d’outils de notre communauté.

# Conflits d’intérêt

Les auteurs déclarent qu’ils n’ont aucun conflit d’intérêt.

# Contribution des auteurs

SAM a écrit le manuscrit et a contribué au devis, au contenu et à l’interprétation de l’activité reportée. IT a contribué aux idées, a facilité un atelier et a révisé le manuscrit. SL a recruté les participants, a facilité un atelier et a révisé le manuscrit. FC est la chercheuse principale, a conçu l’étude et les instruments de la collecte de données et a été impliquée dans la rédaction préliminaire du manuscrit. Le groupe de travail des *Guides de rue* a contribué au devis et à la réalisation de l’activité décrite. Tous les auteurs ont lu et approuvé le manuscrit.

# Appendice

### Appendice 1 – *Les Guides de rue*

*Les* *Guides de rue* est un projet de recherche-action d’une durée de trois ans (2005-2008). Il implique un travail conjoint avec des membres de la communauté utilisatrice de drogues par injection de la ville de Québec, Point de Repères (programme d’échange de seringues local) et des chercheurs de l’Université Laval. Cette collaboration vise à développer un réseau de pairs aidants au sein de la communauté. Le projet comporte deux phases : le renforcement des capacités d’intervention des pairs aidants et les « *intraventions* » de ces pairs aidants.

La phase d’intervention a débuté quand des personnes consommatrices de drogues par injection et reconnues par des intervenants de Point de Repères comme pouvant être intéressées à aider d’autres personnes ont été approchées par l’équipe de recherche. Ces personnes ont été interrogées et invitées à participer. Dix-sept personnes ont pris cet engagement. Leur première tâche a été d'identifier leurs besoins de formation. Elles ont ensuite classé ces besoins par ordre décroissant d'importance. Le consensus auquel elles sont arrivées est le suivant: 1) les premiers soins et la RCR en cas de surdose, 2) la relation d’aide, 3) les soins de la peau et les meilleures pratiques pour éviter les infections de la peau, 4) les aspects juridiques touchant le rôle des pairs aidants, 5) les ressources disponibles dans la communauté pour les personnes qui s'injectent des drogues, 6) les effets des drogues (en particulier les nouvelles drogues et leurs interactions), 7) la façon de gérer sa place et 8) le VIH et le VHC.

Tous ces sujets ont été ensuite abordés dans une série de huit ateliers consécutifs. Les ateliers, qui ont eu lieu sur une base mensuelle, ont été co-préparés par deux ou trois membres du groupe intéressés par un sujet donné, un travailleur de Point de Repères et deux ou trois membres de l'équipe de recherche. L’atelier devait être pratique, accessible et fondé sur des données probantes. Le format a été adapté au mode de vie et aux besoins des participants. À cet effet, les ateliers ont toujours été précédés d'un bon repas chaud, ont eu lieu chaque mois de 17h00 à 19h00 et les contenus abordés ont été consignés dans des documents synthétiques distribués à chaque participant.

Cette première phase de renforcement des capacités vient de se terminer et nous sommes maintenant dans la phase « *intravention* » du projet. Les pairs aidants utilisent maintenant leurs compétences nouvellement développées et leurs connaissances pour interagir avec leurs pairs dans leurs milieux de vie. Ils le font à leur propre rythme et adoptent une approche de réduction des méfaits. Ils gardent des contacts réguliers avec le travailleur de Point de Repères et l’équipe de recherche.

# Remerciements

Le participant dont le pétri d’agar est montré a donné son consentement pour la publication de l’étude. Annick DesCormiers a grandement contribué à la réalisation de l’activité en fournissant l’aide technique nécessaire durant les ateliers et en recueillant les données de croissance microbienne. Nous adressions un merci tout spécial aux travailleurs de Point de Repères. Ce projet a été financé par une subvention accordée à FC par les Instituts de recherche en santé du Canada (IRSC).

# Références

# Figures

##
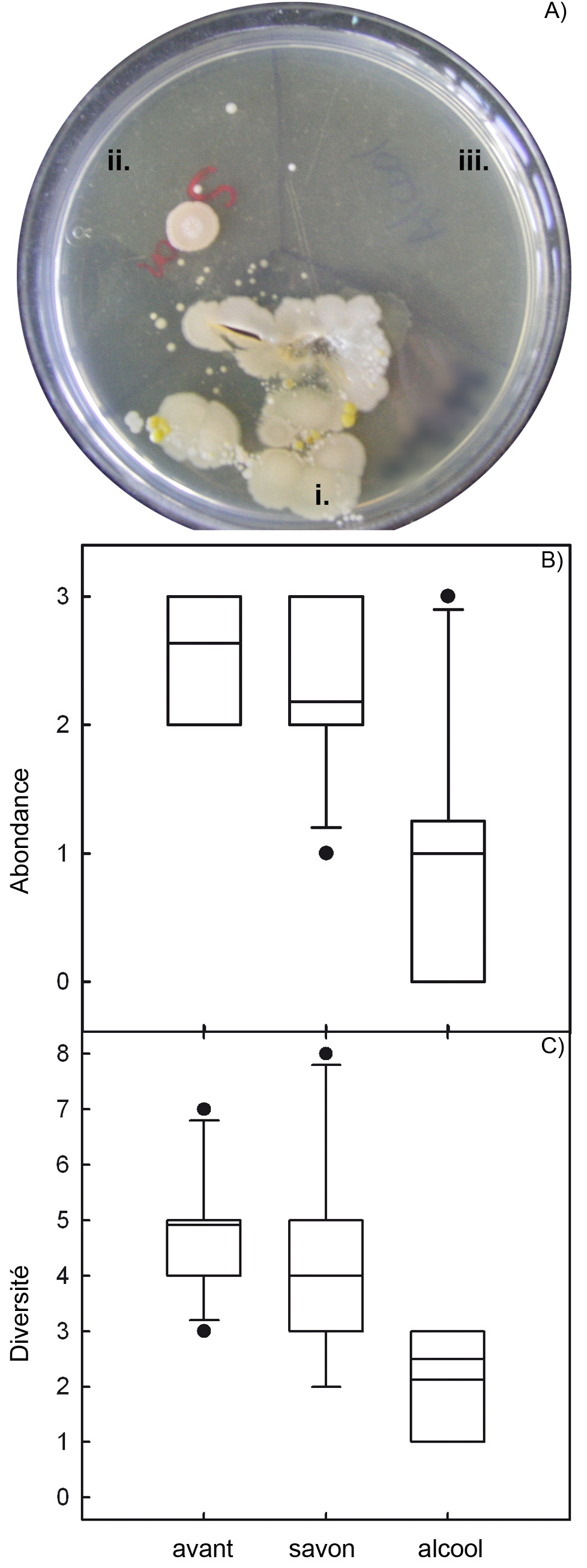
Figure 1  - Microbes de la peau

Les géloses ont été inoculées par le pouce des participants i) avant de le laver, ii) après l’avoir lavé avec un savon antibiotique et de l’eau tiède pendant 30 secondes, iii) après l’avoir frotté avec un tampon d’alcool. **A**) Gélose où l’alcool a été utilisé efficacement. **B**) Boîte à moustaches montrant la quantité de microbes exprimée avec un score catégoriel (3:>300 colonies, 2:50-300 colonies, 1:<50 colonies). L’abondance ne différaient pas après le lavage au savon (*p*=0,13), mais était réduite par l’usage du tampon d’alcool après le lavage des mains (*p*=0,03). **C**) La diversité microbienne est exprimée comme le nombre de colonies visuellement différentes. La diversité ne différait pas après le lavage des mains au savon (*p*=0.16), mais était réduite par l’utilisation du tampon d’alcool suivant le lavage des mains (*p*=0.02).

# Fichiers additionnels

### Fichier additionnel 1 – Article en Français

Format du fichier : DOC

Titre : Article en Français (article in French)

Description : Une version française de l’article a été préparée par les auteurs. Elle est disponible à partir du site Web du *Harm Reduction Journal*.

### Fichier additionnel 2 – Atelier 1 (photographie)

Format du fichier : JPEG

Titre : Workshop 1 (photograph)

Description : Une participante fait la démonstration de la manière dont elle utilise l’eau de Javel pour nettoyer une seringue et une aiguille tel qu’elle le fait lorsqu’elle n’a pas accès à du matériel stérile.

### Fichier additionnel 3 – Atelier 2, partie 1 (photographie)

Format du fichier : JPEG

Titre : Workshop 2, part 1 (photograph)

Description : Installation du microscope branché à un écran de télévision. Les participants pouvaient y voir des objets microscopiques provenant d’échantillons de l’environnement immédiat.

### Fichier additionnel 4 – Atelier 2, partie 2 (photographie)

Format du fichier : JPEG

Titre : Workshop 2, part 2 (photograph)

Description : **A**) Une participante prélève un échantillon au niveau du pli de son coude. **B**) Croissance microbienne obtenue à partir de cet échantillon.

### Fichier additionnel 5 – Atelier 3 (photographie)

Format du fichier : JPEG

Titre : Workshop 3 (photograph)

Description : **A**) Installation utilisée pour la démonstration du lavage des mains. **B**) Les mains d’un participant vues sous la lampe UV. De la poudre fluorescente n’ayant pas été délogée par le lavage des mains est visible au bout de ses doigts.
