# Supplementary figures and images for: Seeing is believing: an educational outreach activity on disinfection practices
Source: Harm Reduct J. 2008 Feb 12;5:7. doi: 10.1186/1477-7517-5-7 (PMC2265699; doi:10.1186/1477-7517-5-7)

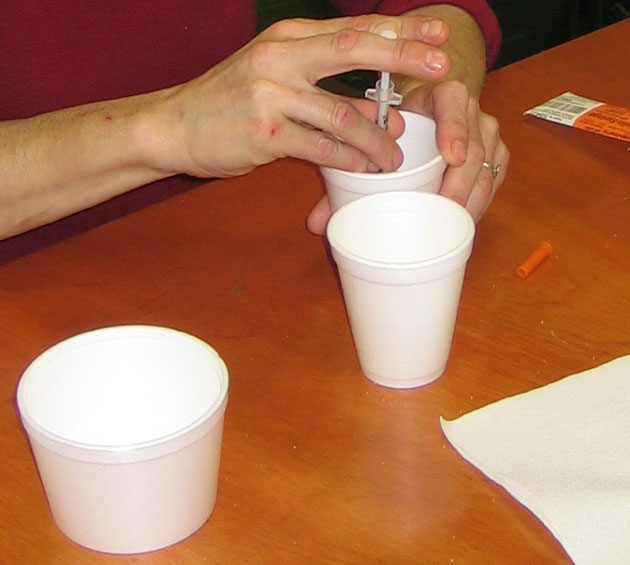

Supplement: Additional File 2 — Workshop 1 (photograph). A participant is demonstrating the way she uses bleach to clean a syringe and needle as she usually does when she does not have access to sterile material. [file 1477-7517-5-7-S2.jpeg]

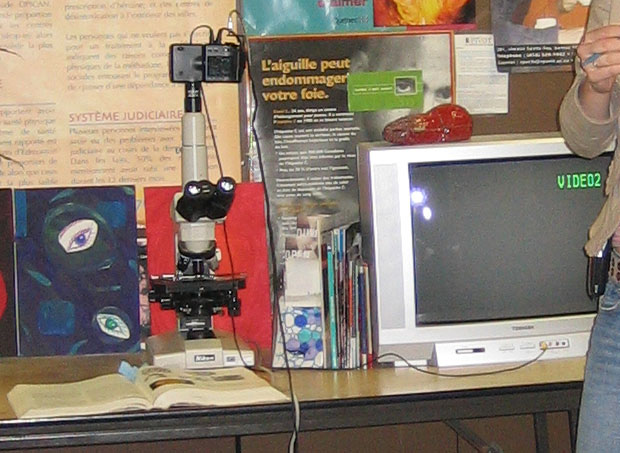

Supplement: Additional File 3 — Workshop 2, part 1 (photograph). A microscope was mounted on a TV-set so that participants could see microscopic objects sampled from the surroundings. [file 1477-7517-5-7-S3.jpeg]

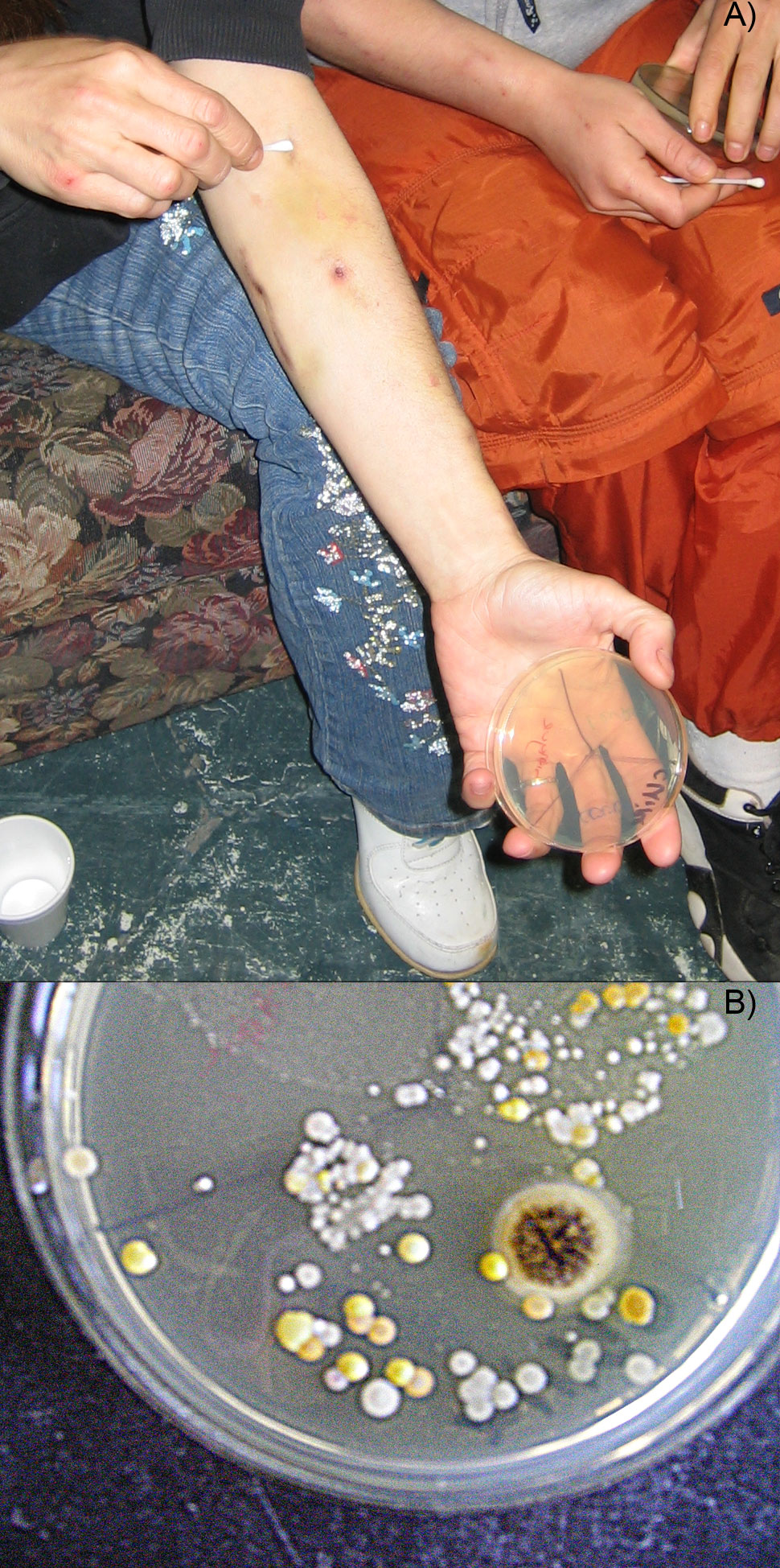

Supplement: Additional File 4 — Workshop 2, part 2 (photograph). A) A participant is taking a sample from her cubital fossa. B) Microbial growth from this sample. [file 1477-7517-5-7-S4.jpeg]

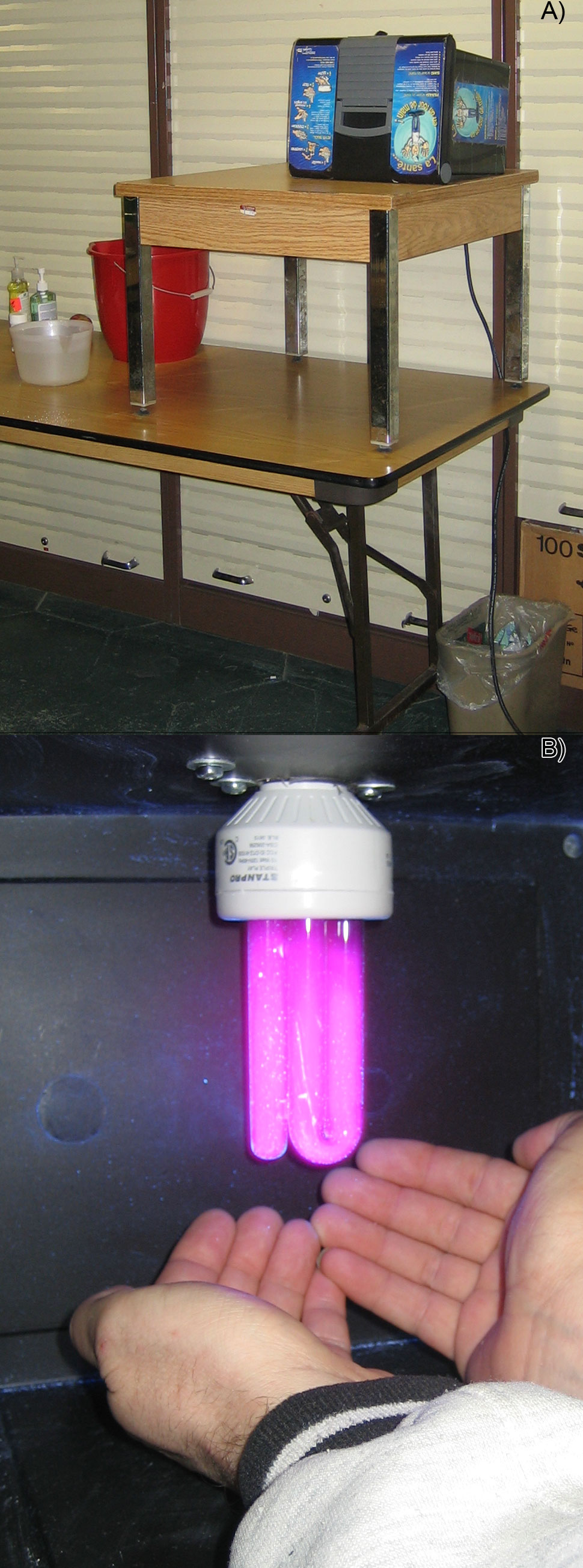

Supplement: Additional File 5 — Workshop 3 (photograph). A) The set-up used to demonstrate hand washing techniques. B) A participant's hands under the UV-lamp. Some fluorescent powder remained after he washed his hand as can be seen on the tips of his fingers. [file 1477-7517-5-7-S5.jpeg]
